# Vital Signs: Cervical Cancer Incidence, Mortality, and Screening — United States, 2007–2012

**Published:** 2014-11-07

**Authors:** Vicki B. Benard, Cheryll C. Thomas, Jessica King, Greta M. Massetti, V. Paul Doria-Rose, Mona Saraiya

**Affiliations:** 1 Division of Cancer Prevention and Control, CDC; 2 Division of Cancer Control and Population Science, NCI

## Abstract

**Background:**

Cervical cancer screening is one of the greatest cancer prevention achievements, yet some women still develop or die from this disease.

**Objective:**

To assess recent trends in cervical cancer incidence and mortality, current screening percentages, and factors associated with higher incidence and death rates and inadequate screening.

**Methods:**

Percentages of women who had not been screened for cervical cancer in the past 5 years were estimated using data from the 2012 Behavioral Risk Factor Surveillance System survey. State-specific cervical cancer incidence data from the United States Cancer Statistics and mortality data from the National Vital Statistics System were used to calculate incidence and death rates for 2011 by state. Incidence and death rates and annual percentage changes from 2007 to 2011 were calculated by state and U.S. Census region.

**Results:**

In 2012, the percentage of women who had not been screened for cervical cancer in the past 5 years was estimated to be 11.4%; the percentage was larger for women without health insurance (23.1%) and for those without a regular health care provider (25.5%). From 2007 to 2011, the cervical cancer incidence rate decreased by 1.9% per year while the death rate remained stable. The South had the highest incidence rate (8.5 per 100,000), death rate (2.7 per 100,000), and percentage of women who had not been screened in the past 5 years (12.3%).

**Conclusions:**

Trends in cervical cancer incidence rates have decreased slightly while death rates have been stable over the last 5 years. The proportion of inadequately screened women is higher among older women, Asians/Pacific Islanders, and American Indians/Alaska Natives.

**Implications for Public Health Practice:**

There continue to be women who are not screened as recommended, and women who die from this preventable cancer. Evidence-based public health approaches are available to increase women’s access to screening and timely follow-up of abnormal results.

## Introduction

Since the introduction and widespread use of the Papanicolaou (Pap) test in the 1950s in the United States, cervical cancer incidence and mortality have decreased dramatically (1,2). In addition to screening with a Pap test alone every 3 years, recent cervical cancer screening recommendations now include the use of the human papillomavirus (HPV) test (used to detect infection with oncogenic HPV types associated with cervical cancers) with the Pap test among women aged 30–65 years every 5 years (1,3). Despite evidence that cervical cancer screening saves lives, the incidence and death rates from cervical cancer remain substantial, especially among populations with limited access to care (4). Over half of all new cases occur in women who have never or rarely been screened (5). Recent findings have reported that uninsured women or those without a regular health care provider were significantly less likely to receive cervical cancer screening (6).

*Healthy People 2020* (HP2020) cervical cancer objectives include increasing screening rates to a target of 93%, reducing the incidence rate to 7.1 per 100,000 women, and reducing the death rate to 2.2 per 100,000 women (available at: http://www.healthypeople.gov). This report presents state-specific screening prevalence data from the 2012 Behavioral Risk Factor Surveillance System (BRFSS) survey, state-specific cervical cancer incidence and death rates for 2007 to 2011 (combined) and 2011 (alone), and annual percentage changes in the incidence and death rates from 2007 to 2011 to examine progress toward these objectives.

**Key Points**

- In 2011 in the United States, 12,109 women developed cervical cancer and 4,092 died.
- Approximately 1 in 10 women aged 21–65 years had not been screened for this preventable disease in the past 5 years.
- Approximately 1 in 4 women ages 21–65 years without health insurance or a regular health care provider had not been screened for cervical cancer in the past 5 years.
- The South had the highest incidence of cervical cancer cases and deaths and the lowest prevalence of screening.
- The greatest impact on current cervical cancer will be to screen women who have not been screened within the past 5 years.

## Methods

The BRFSS survey is a state-based, random-digit–dialed telephone survey of the civilian, noninstitutionalized adult population of the United States that collects information on health risk behaviors, preventive health practices, and health care access in the United States (available at http://www.cdc.gov/brfss). Survey data were available for all 50 states and the District of Columbia (DC) in 2012 with a median survey response rate of 49.7%.

Female BRFSS respondents were asked about having a Pap test (“A Pap test is a test for cancer of the cervix. Have you ever had a Pap test?”) and when this test was last performed. For this study, it was impossible to determine whether a woman was screened with both a Pap and HPV test (co-test) because HPV testing questions were not collected in the 2012 BRFSS survey. Because screening intervals vary depending on the type of test, and to include women who might have been screened with a co-test, respondents were categorized as not screened in the past 5 years if they reported not having had a Pap test at all or in the past 5 years. For consistency with current screening recommendations (1,3), analyses were restricted to women aged 21–65 years who reported not having had a hysterectomy. For analysis by age, women aged 21–22 years, who might not have had an opportunity to get screened within the first year of the recommended screening age, were excluded (1,3). Respondents who refused to answer or answered “don’t know/not sure” were excluded. BRFSS data were weighted using advanced raking techniques (7).

United States Cancer Statistics (USCS) (available at http://www.cdc.gov/uscs) provide official federal cancer incidence statistics in each state, using data from the National Program of Cancer Registries and the Surveillance, Epidemiology, and End Results (SEER) Program. Forty-nine states and DC met USCS publication criteria for the period 2007–2011, representing 99.1% of the U.S. population. Incident cervical cancers were coded according to the *International Classification of Disease for Oncology, Third Edition*.

Cancer mortality statistics are based on all death certificates filed in the 50 states and DC, covering 100% of the U.S. population. The mortality data are provided by the National Center for Health Statistics. All reported deaths with cervical cancer identified as the underlying cause of death according to the *International Classification of Diseases, Tenth Revision* during 2007–2011 were included.

Incidence and death rates for 2007 to 2011 (combined) and 2011 (alone) and trend analyses for the period 2007–2011 were conducted. Population estimates by sex, age group, and race/ethnicity were from the U.S. Census, as modified by SEER (available at http://www.seer.cancer.gov/popdata).

Screening, incidence, and mortality data were age-adjusted to the 2000 U.S. standard population by the direct method. Incidence and mortality data reflect 99.1% and 100% of the population, not samples. However, to be able to compare rates among states, 95% confidence intervals (CIs) were calculated using the Tiwari method (8). Rates and annual percentage changes (APCs) were calculated for all races/ethnicities, and all age groups combined for each state and U.S. Census region (available at https://www.census.gov/geo/reference/gtc/gtc_census_divreg.html).

## Results

The 2012 BRFSS survey was administered to 133,851 women aged 21–65 years who had complete Pap data and no hysterectomy, representing 70,462,535 women in the United States. Of these 70 million women, an estimated 8.2 million (11.4%) had not been screened for cervical cancer in the past 5 years, with higher percentages among women aged 23–29 years (13.4%), 60–65 years (12.6%), Asians/Pacific Islanders (19.7%), and American Indians/Alaska Natives (16.5%). Among women with no health insurance, 23.1% had not been screened in the past 5 years, including higher percentages among women aged 50–59 years (29.8%) and Asians/Pacific Islanders (32.5%) (Table 1). Among women with no regular health care provider, 25.5% had not been screened in the past 5 years, with the highest percentages among those aged 60–65 years (37.1%) and Asians/Pacific Islanders (40.8%).

During 2007–2011, there were 62,150 cervical cancer cases in the United States. From 2007 to 2011, age-adjusted cervical cancer incidence rates decreased significantly overall (1.9% per year) and in Arizona, California, Georgia, New York, and Rhode Island, which reported the largest annual percentage decrease (9.9%) (Table 2). Compared with other Census regions, the South had the highest incidence rate (8.5 per 100,000) (Table 2). In 2011, the overall U.S. incidence rate was 7.5 per 100,000 women (12,109 new cases), ranging from 4.5 in New Hampshire to 13.7 in DC (Figure).

During 2007–2011, there were 19,969 cervical cancer deaths in the United States. The overall age-adjusted cervical cancer death rate remained stable (nonsignificant APC of −1.2% per year), but significantly decreased in two states from 2007 to 2011 (North Carolina, 4.1%, and Virginia, 11.5%) (Table 2). Compared with other Census regions, the South had the highest death rate (2.7 per 100,000) (Table 2). In 2011, the overall U.S. death rate was 2.3 per 100,000 women (4,092 deaths), ranging from 1.2 in Utah to 4.8 in West Virginia (Figure).

## Conclusion and Comments

Important disparities persist in cervical cancer screening, incidence, and mortality. While overall cervical cancer death rates have remained stable in the United States, incidence rates declined 1.9% per year. By state, incidence rates were stable across most states, with five having a significant decrease. Incidence and death rates for the United States have remained above the HP2020 targets, but are close to reaching them. Previous data from a national survey has shown that 83% of women were up-to-date with current cervical cancer recommendations with a slight downward trend observed in the percentage of women screened during 2008–2010 (6). More progress needs to be made toward the HP2020 objective for cervical cancer screening, especially among women who lack access to health care because they lack health care coverage or a regular health care provider. The findings show that approximately 1 in 10 women had not been screened in the past 5 years, including 1 in 4 women who had no health insurance and 1 in 4 who had no regular health care provider.

Disparities by age, race/ethnicity, and geography exist in cervical cancer. Whereas younger and older women had comparable rates of not having been screened in the past 5 years, developing or dying from cervical cancer is rare in younger women (9). More concerning is higher percentages of inadequately screened women among those aged >40 years, who have the highest rates of cervical cancer incidence and death. Cervical cancer incidence rates are higher for black and Hispanic women than for white women, and death rates are higher for black women (available at http://www.cdc.gov/uscs). Higher incidence and death rates and percentages of not having been screened in the past 5 years were reported in the South compared with other Census regions. The findings regarding geographic differences support other studies with findings pertaining to Appalachia, southeastern Atlantic states, the lower Mississippi Valley, and along the United States–Mexico border (10,11).

Financial and nonfinancial barriers might explain some disparities in screening percentages. Of the estimated 8.2 million women who had not been screened in the past 5 years, 69.9% had insurance and had a regular health care provider, 9.6% had insurance but no regular health care provider, 9.8% had no insurance but did have a regular health care provider, and 10.7% had neither. For more than 20 years, the National Breast and Cervical Cancer Early Detection Program (available at http://www.cdc.gov/cancer/nbccedp) has provided free or low-cost screening and diagnostic breast and cervical cancer services to low-income, underinsured, and uninsured women and access to state Medicaid programs for treatment. In addition, the Affordable Care Act is reducing financial barriers to screening by increasing access to insurance coverage for clinical preventive services rated A or B by the U.S. Preventive Services Task Force. Cervical cancer screening is now provided with no cost-sharing for women covered by Medicare and in most private insurance plans and for newly eligible beneficiaries of the Medicaid expansion (12). Both could help in the effort to increase the cervical cancer screening proportion from 83% in 2010 to the HP2020 target of 93% (6). However, nonfinancial barriers, such as lack of awareness and lack of transportation also need to be addressed (13).

In addition to focusing on women who have not been screened in the past 5 years, continued timely and regular screening for women who are meeting current cervical cancer screening recommendations must continue. In 2012, for the first time, all national screening organizations (the U.S. Preventive Services Task Force, American Cancer Society, and American College of Obstetrics and Gynecology) agreed on when and how often to screen for cervical cancer (available at http://www.cdc.gov/cancer/cervical/pdf/guidelines.pdf). With multiple age-dependent options for screening and evolving technologies, there is a continuing need to clarify for providers and for women the best approach for screening.

The introduction of the HPV vaccine as a primary prevention measure to reduce cervical cancer cases and deaths is promising, but the vaccine continues to be underused. The Advisory Committee on Immunization Practices recommends routine HPV vaccination of children aged 11 or 12 years (14). Findings from the 2013 National Immunization Survey-Teen indicate that 37.6% of adolescent girls (aged 13–17 years) and 13.9% of adolescent boys completed the 3-dose series (15). Modeling studies have shown that HPV vaccination and cervical cancer screening combined could prevent nearly 93% of new cervical cancer cases (16). Efforts are needed to improve HPV vaccination as recommended. Current cervical cancer screening recommendations remain the same, regardless of vaccination status (available at http://www.cdc.gov/cancer/cervical/pdf/guidelines.pdf).

The findings in this report are subject to at least five limitations. First, because BRFSS is administered by telephone, only noninstitutionalized adults with landline telephones or cell phones are represented and might not be representative of the entire U.S. population. Second, recent trends in cervical cancer screening cannot be examined because of changes in BRFSS sampling methodology and weighting in 2011 (7). Third, responses regarding screening are self-reported and not confirmed by review of medical records. Fourth, the screening prevalence data included women without a hysterectomy; however, incidence rates did not adjust for hysterectomy and might be underreported (17). Finally, because the BRFSS median response rate was <50%, nonresponse bias might have affected the results.

A more thorough understanding of the etiologic role of HPV in cervical cancer has provided the foundation for targeted approaches for prevention, including the HPV vaccination and HPV-based screening. However, regardless of the improvement in prevention methods, most cervical cancer occurs in women who have not had recent screening. By addressing financial and nonfinancial barriers, there is the opportunity to see progress by increasing screening and reducing incidence and death from this disease.

## Figures and Tables

**FIGURE:**
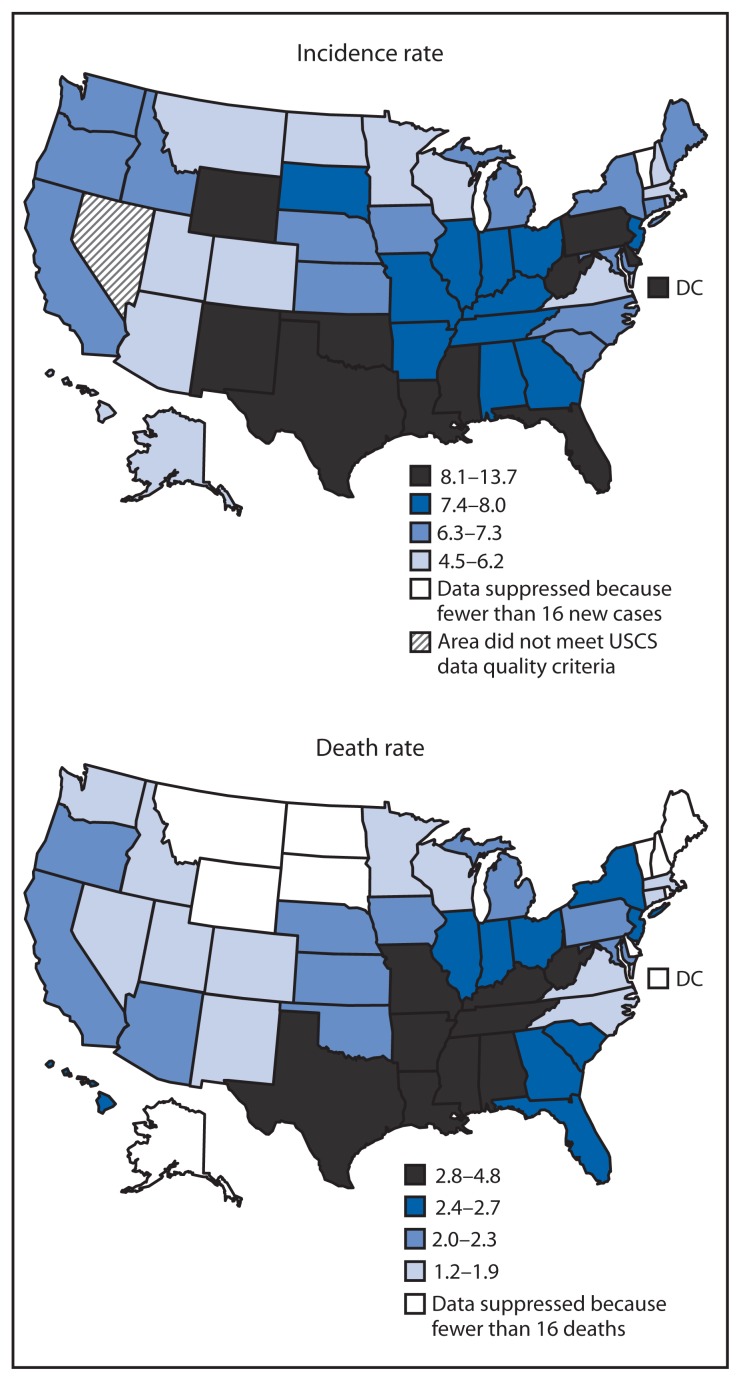
Cervical cancer incidence and death rates*— United States, 2011 **Abbreviation:** USCS = U.S. Cancer Statistics. **Sources:** Cancer incidence combines cancer registry data from the National Program of Cancer Registries and the Surveillance, Epidemiology, and End Results Program that met USCS publication criteria for 2011, covering 99.1% of the U.S. population. Additional information available at: http://www.cdc.gov/uscs. Mortality data are provided by the National Vital Statistics System, covering 100% of the U.S. population. * Per 100,000 population, age-adjusted to the 2000 US standard population (19 age groups).

**TABLE 1:** Percentage of women aged 21–65 years who had not been screened for cervical cancer in the past 5 years,* by age group and race/ethnicity — Behavioral Risk Factor Surveillance System, United States, 2012

	Overall % not screened in the past 5 years	% with no health insurance not screened in the past 5 years	% with no regular health care provider not screened in the past 5 years
**Overall**	**11.4**	**23.1**	**25.5**
**Age group (yrs)** †			
23–29	13.4	19.1	19.7
30–39	8.3	16.6	17.2
40–49	10.1	23.9	26.1
50–59	11.7	29.8	33.7
60–65	12.6	26.6	37.1
**Race/Ethnicity**			
White	10.8	28.8	27.7
Black	9.2	16.8	21.4
A/PI	19.7	32.5	40.8
AI/AN	16.5	26.9	29.2
Other	13.8	29.7	34.2
Hispanic	11.7	16.7	18.4

**Abbreviations:** A/PI = Asian/Pacific Islander; AI/AN = American Indian/Alaska Native.

* Percentage of women aged 21–65 years who reported not having a hysterectomy and not receiving a Papanicolaou (Pap) at all or in the past 5 years; age-standardized to the 2000 US Census standard population.

† Data are presented for 23–65 year olds because women aged 21–22 years might not have had the opportunity for screening in the first year of that recommendation.

**TABLE 2:** Age-adjusted cervical cancer incidence and death rates* (2007–2011), annual percentage change (APC)† from 2007 to 2011, and percentage of women aged 21–65 years in 2012 not screened for cervical cancer in the past 5 years,§ by Census region and state — United States

	Incidence rate				Death rate				% overall not screened		% with no insurance not screened		% with no provider not screened	
					
	2007–2011				2007–2011				2012					
			
Census region/State	Rate	(95% CL)	APC	(95% CL)	Rate	(95% CL)	APC	(95% CL)	%	(95% CI)	%	(95% CI)	%	(95% CI)
**United States overall**	**7.8**	**(7.8, 7.9)**	−**1.9**¶	**(**−**3.5,** −**0.3)**	**2.3**	**(2.3, 2.4)**	−**1.2**	**(**−**3.3, 0.9)**	**11.4**	**(11.1–11.8)**	**23.1**	**(22.0–24.3)**	**25.5**	**(24.3–26.7)**
**Census region**														
Northeast	7.5	(7.3, 7.6)	−2.7¶	(−4.8, −0.6)	2.1	(2.0, 2.1)	0.4	(−4.0, 5.0)	10.9	(10.1–11.9)	22.6	(19.7–25.8)	28.0	(24.5–31.9)
Midwest	7.4	(7.3, 7.5)	−1.2	(−3.3, 1.0)	2.2	(2.2, 2.3)	−0.6	(−3.2, 2.0)	10.6	(10.0–11.2)	25.9	(23.7–28.2)	28.1	(25.9–30.4)
South	8.5	(8.4, 8.6)	−1.4	(−3.6, 0.8)	2.7	(2.6, 2.7)	−1.9	(−4.5, 0.7)	12.3	(11.6–12.9)	23.8	(22.1–25.6)	25.4	(23.6–27.4)
West	7.3	(7.2, 7.5)	−2.8¶	(−4.7, −0.8)	2.1	(2.0, 2.2)	−1.8	(−3.7, 0.3)	11.5	(10.7–12.4)	20.6	(18.6–22.7)	23.3	(21.1–25.8)
**State**														
Alabama	8.6	(8.0, 9.1)	−4.3	(−11.9, 3.8)	3.0	(2.8, 3.4)	1.4	(−6.3, 9.7)	12.5	(10.8–14.5)	27.7	(22.7–33.4)	26.9	(21.5–32.9)
Alaska	7.1	(5.8, 8.5)	−8.2	(−33.2, 26.2)	2.4	(1.6, 3.3)	—**	—	12.4	(10.0–15.4)	21.6	(15.8–28.8)	23.3	(18.2–29.3)
Arizona	6.9	(6.5, 7.4)	−4.9¶	(−9.4, −0.2)	2.0	(1.8, 2.2)	2.2	(−3.1, 7.9)	13.8	(11.5–16.6)	22.9	(17.5–29.4)	23.3	(17.8–29.9)
Arkansas	10.0	(9.3, 10.7)	−3.8	(−14.9, 8.7)	3.4	(3.0, 3.8)	0.8	(−10.9, 14.1)	15.9	(13.4–18.7)	27.4	(22.2–33.4)	32.8	(26.5–39.7)
California	7.8	(7.7, 8.0)	−3.8¶	(−6.2, −1.4)	2.3	(2.2, 2.4)	−1.0	(−5.9, 4.2)	10.5	(9.0–12.1)	17.8	(14.6–21.6)	20.7	(17.0–25.0)
Colorado	6.2	(5.8, 6.6)	−3.9	(−8.3, 0.7)	1.6	(1.4, 1.9)	−6.2	(−24.2, 16.1)	9.3	(8.1–10.7)	22.0	(18.0–26.5)	25.4	(21.3–30.0)
Connecticut	6.2	(5.7, 6.8)	1.6	(−10.7, 15.5)	1.6	(1.3, 1.8)	3.2	(−6.8, 14.3)	8.6	(7.2–10.3)	24.1	(18.1–31.3)	28.3	(22.2–35.4)
Delaware	8.8	(7.6, 10.1)	−0.3	(−2.8, 2.2)	2.5	(1.9, 3.3)	—	—	7.3	(5.8–9.2)	18.6	(13.3–25.5)	23.7	(16.1–33.5)
District of Columbia	10.3	(8.7, 12.1)	3.7	(−20.3, 34.8)	2.6	(1.8, 3.5)	—	—	8.8	(6.3–12.2)	13.3	(6.0–27.0)	14.7	(8.5–24.3)
Florida	9.0	(8.8, 9.3)	−0.9	(−5.8, 4.3)	2.6	(2.5, 2.8)	2.4	(−2.3, 7.4)	14.7	(12.4–17.5)	29.0	(23.4–35.4)	26.8	(21.7–32.5)
Georgia	8.2	(7.8, 8.5)	−3.4¶	(−5.8, −0.9)	2.7	(2.5, 2.9)	−3.7	(−7.6, 0.3)	10.9	(9.0–13.2)	22.6	(17.6–28.5)	23.0	(17.6–29.5)
Hawaii	7.3	(6.4, 8.3)	−4.7	(−23.3, 18.3)	1.8	(1.4, 2.3)	—	—	13.0	(11.1–15.2)	25.0	(18.5–32.8)	27.6	(21.5–34.6)
Idaho	5.9	(5.1, 6.7)	9.7	(−5.0, 26.7)	2.1	(1.7, 2.6)	—	—	18.7	(15.6–22.3)	26.0	(19.5–33.6)	32.2	(24.8–40.6)
Illinois	8.4	(8.1, 8.7)	−3.5	(−10.7, 4.3)	2.6	(2.5, 2.8)	−1.1	(−5.9, 4.0)	9.4	(7.8–11.4)	17.8	(12.2–25.2)	26.8	(19.7–35.3)
Indiana	7.5	(7.1, 8.0)	0.0	(−1.9, 2.0)	2.4	(2.2, 2.6)	1.7	(−10.8, 16.0)	14.3	(12.5–16.3)	35.7	(30.1–41.8)	38.7	(32.8–45.0)
Iowa	6.8	(6.2, 7.4)	1.5	(−5.5, 9.0)	2.1	(1.8, 2.4)	−0.4	(−6.3, 5.8)	9.5	(8.0–11.3)	25.1	(18.8–32.8)	25.3	(19.4–32.3)
Kansas	7.2	(6.5, 7.8)	3.5	(−11.5, 21.1)	1.9	(1.6, 2.2)	−0.1	(−8.8, 9.3)	11.4	(10.0–12.9)	25.4	(21–30.4)	28.3	(23.3–34)
Kentucky	8.7	(8.1, 9.3)	−2.0	(−8.4, 4.7)	3.1	(2.8, 3.4)	1.5	(−13.5, 19.1)	13.6	(11.9–15.5)	25.2	(20.8–30.3)	27.9	(22.9–33.5)
Louisiana	9.4	(8.9, 10.0)	−2.2	(−11.8, 8.5)	3.1	(2.8, 3.5)	−4.5	(−14.7, 6.8)	12.1	(10.2–14.3)	20.5	(16.2–25.5)	28.7	(22.8–35.3)
Maine	6.8	(5.9, 7.7)	−0.8	(−10.4, 9.9)	1.6	(1.2, 2.0)	—	—	6.9	(5.8–8.2)	18.8	(14.4–24.1)	34.6	(27.5–42.4)
Maryland	6.8	(6.4, 7.2)	0.8	(−6.4, 8.6)	2.2	(2.0, 2.5)	−5.2	(−11.0, 1.1)	8.7	(7.2–10.4)	18.8	(13.5–25.6)	16.2	(11.9–21.6)
Massachusetts	5.5	(5.1, 5.8)	−0.3	(−3.8, 3.3)	1.4	(1.2, 1.6)	6.2	(−9.6, 24.8)	7.8	(6.9–8.9)	19.6	(14.0–26.7)	22.7	(18.1–27.9)
Michigan	7.3	(6.9, 7.6)	−3.8	(−8.4, 1.0)	2.1	(1.9, 2.3)	2.1	(−1.8, 6.0)	9.8	(8.5–11.2)	26.9	(22.0–32.5)	30.7	(25.5–36.5)
Minnesota	6.0	(5.6, 6.4)	1.8	(−2.9, 6.7)	1.4	(1.2, 1.6)	1.2	(−19.1, 26.5)	8.4	(7.1–9.9)	19.2	(14.4–25.2)	19.6	(16.0–23.7)
Mississippi	9.7	(9.0, 10.4)	1.5	(−7.3, 11.2)	3.5	(3.1, 3.9)	−8.1	(−16.7, 1.3)	14.9	(13.0–17.1)	26.4	(21.6–31.8)	26.0	(21.1–31.6)
Missouri	8.1	(7.7, 8.6)	−0.7	(−7.7, 6.8)	2.5	(2.3, 2.8)	0.9	(−12.4, 16.2)	13.1	(11.1–15.3)	32.3	(26.0–39.3)	26.9	(21.2–33.4)
Montana	6.3	(5.3, 7.3)	1.1	(−7.4, 10.4)	1.5	(1.1, 2.0)	—	—	11.6	(10.0–13.4)	24.2	(19.7–29.4)	23.9	(19.8–28.6)
Nebraska	7.2	(6.4, 8.0)	−1.6	(−16, 15.4)	1.9	(1.5, 2.3)	—	—	11.1	(10.0–12.3)	21.9	(18.2–26.2)	26.8	(22.4–31.8)
Nevada	NS	NS	NS	NS	2.1	(1.8, 2.5)	−4.3	(−24.3, 20.9)	17.7	(15.1–20.6)	28.0	(22.5–34.3)	29.0	(23.4–35.2)
New Hampshire	5.2	(4.4, 6.0)	−4.6	(−13.0, 4.6)	1.8	(1.4, 2.3)	—	—	8.6	(7.0–10.6)	26.3	(20.2–33.5)	32.7	(25.4–41.0)
New Jersey	8.3	(8.0, 8.7)	−4.2	(−9.0, 0.9)	2.3	(2.1, 2.5)	6.0	(−0.4, 12.8)	11.8	(10.4–13.3)	22.9	(19.2–27.1)	25.7	(21.4–30.6)
New Mexico	7.6	(6.8, 8.4)	2.2	(−7.5, 13.0)	2.1	(1.7, 2.5)	−12.6	(−29.9, 9.0)	12.0	(10.5–13.6)	23.6	(19.9–27.9)	22.5	(18.9–26.5)
New York	8.1	(7.9, 8.4)	−3.8¶	(−6.8, −0.8)	2.3	(2.2, 2.4)	−2.1	(−11.4, 8.1)	12.0	(10.0–14.3)	20.3	(15.0–26.9)	26.6	(20.2–34.0)
North Carolina	7.0	(6.7, 7.3)	−0.8	(−5.8, 4.3)	2.1	(1.9, 2.3)	−4.1¶	(−7.7, −0.3)	9.6	(8.5–10.9)	21.8	(18.5–25.6)	25.6	(21.6–30.0)
North Dakota	6.2	(5.0, 7.6)	—	—	1.3	(0.9, 2.0)	—	—	10.2	(8.1–12.7)	22.4	(15.6–31.1)	26.5	(19.7–34.5)
Ohio	7.7	(7.4, 8.0)	−0.7	(−7.3, 6.3)	2.6	(2.5, 2.8)	−3.5	(−7.8, 0.9)	11.0	(9.7–12.5)	26.1	(21.8–31.0)	30.6	(25.8–35.8)
Oklahoma	9.9	(9.3, 10.6)	−2.2	(−9.7, 5.9)	2.8	(2.5, 3.2)	−5.4	(−21.9, 14.5)	14.0	(12.4–15.9)	26.0	(21.9–30.5)	30.9	(26.4–35.8)
Oregon	7.2	(6.7, 7.8)	−7.1	(−14.2, 0.5)	2.0	(1.7, 2.3)	3.4	(−16.5, 27.9)	12.0	(10.0–14.4)	22.9	(17.6–29.2)	35.6	(28.9–42.9)
Pennsylvania	7.9	(7.6, 8.2)	−1.6	(−8.0, 5.3)	2.1	(2.0, 2.3)	0.8	(−7.9, 10.3)	11.9	(10.4–13.6)	26.8	(22.0–32.1)	33.1	(27.2–39.6)
Rhode Island	6.2	(5.3, 7.2)	−9.9¶	(−15.7, −3.8)	1.4	(1.0, 1.9)	—	—	7.8	(6.2–9.7)	15.7	(11.3–21.6)	29.0	(22.1–37.0)
South Carolina	8.2	(7.7, 8.8)	−2.5	(−15.1, 12.0)	2.8	(2.6, 3.2)	−4.3	(−9.4, 1.1)	12.7	(11.1–14.4)	26.5	(22.3–31.2)	30.7	(26.0–35.7)
South Dakota	6.6	(5.5, 7.9)	5.5	(−9.4, 22.8)	2.1	(1.5, 2.8)	—	—	10.4	(8.3–12.9)	29.3	(21.9–38.1)	22.0	(16.1–29.1)
Tennessee	8.5	(8.1, 9.0)	−0.2	(−9.1, 9.5)	2.8	(2.6, 3.1)	1.7	(−10.5, 15.5)	11.4	(9.6–13.4)	24.2	(19.4–29.9)	28.1	(22.8–34.2)
Texas	9.4	(9.1, 9.6)	−1.5	(−3.6, 0.5)	2.8	(2.7, 2.9)	−2.2	(−6.1, 1.7)	13.7	(12.0–15.6)	21.6	(18.3–25.4)	23.7	(19.8–28.1)
Utah	5.3	(4.7, 5.9)	4.0	(−5.3, 14.2)	1.2	(0.9, 1.5)	—	—	14.0	(12.6–15.4)	22.0	(18.1–26.3)	27.1	(23.3–31.4)
Vermont	4.3	(3.3, 5.4)	—	—	1.3	(0.8, 1.9)	—	—	8.6	(7.0–10.4)	30.9	(22.8–40.5)	24.4	(17.7–32.6)
Virginia	6.3	(5.9, 6.6)	−1.6	(−4.0, 0.8)	2.1	(1.9, 2.3)	−11.5¶	(−18.0, −4.6)	9.0	(7.6–10.6)	19.9	(15.3–25.4)	17.7	(13.7–22.5)
Washington	6.9	(6.5, 7.3)	3.3	(−4.4, 11.6)	1.9	(1.7, 2.1)	−5.0	(−17.7, 9.8)	11.1	(9.8–12.5)	23.2	(19.5–27.4)	25.4	(21.6–29.6)
West Virginia	10.2	(9.3, 11.2)	3.2	(−4.4, 11.5)	3.3	(2.8, 3.8)	11.1	(−10.4, 37.7)	14.3	(12.2–16.6)	26.5	(21.1–32.6)	28.3	(22.7–34.7)
Wisconsin	5.9	(5.5, 6.3)	1.6	(−5.6, 9.4)	1.5	(1.4, 1.8)	−1.9	(−14.2, 12.1)	9.3	(7.3–11.9)	23.4	(15.6–33.6)	26.1	(18.4–35.6)
Wyoming	8.4	(6.9, 10.1)	11.0	(−2.0, 25.7)	2.8	(2.0, 3.8)	—	—	14.1	(11.6–17.2)	23.0	(17.6–29.6)	25.1	(19.3–32.0)

**Abbreviations:** CL = confidence limits; CI = confidence interval; NS = not shown; state did not meet US Cancer Statistics (USCS) publication criteria for 2007–2011.

**Sources:** Cancer incidence combines cancer registry data from the National Program of Cancer Registries and the Surveillance, Epidemiology, and End Results Program that met USCS publication criteria for 2007–2011, covering 99.1% of the U.S. population. Additional information available at http://www.cdc.gov/uscs. Mortality data are provided by the National Vital Statistics System, covering 100% of the U.S. population. Cervical cancer screening data are from the 2012 Behavioral Risk Factor Surveillance survey. Available at http://www.cdc.gov/brfss.

* Per 100,000 population, age-adjusted to the 2000 US standard population (19 age groups).

† Calculated using weighted least squares method and joinpoint regression modeling.

§ Percentage of women aged 21–65 years who reported not having a hysterectomy and not receiving a Papanicolaou (Pap) at all or in the past 5 years; age-standardized to the 2000 US standard population.

¶ The APC is significantly different from zero (p<0.05).

** Data suppressed because there were fewer than 16 cases or deaths in a single year.
